# Antioxidant Effects of the Ethanol Extract from Flower of *Camellia japonica* via Scavenging of Reactive Oxygen Species and Induction of Antioxidant Enzymes

**DOI:** 10.3390/ijms12042618

**Published:** 2011-04-18

**Authors:** Mei Jing Piao, Eun Sook Yoo, Young Sang Koh, Hee Kyoung Kang, Junoh Kim, Yong Jin Kim, Hak Hee Kang, Jin Won Hyun

**Affiliations:** 1 School of Medicine and Applied Radiological Science Research Institute, Jeju National University, Jeju 690–756, Korea; E-Mails: mjpiao@hanmail.net (M.J.P.); eunsyoo@jejunu.ac.kr (E.S.Y.); yskoh7@jejunu.ac.kr (Y.S.K.); pharmkhk@jejunu.ac.kr (H.K.K.); 2 Amore Pacific Co. R&D Center, Yongin-si, Gyeonggi-do 446–729, Korea; E-Mails: junoh@amorepacific.com (J.K.); jaykim@amorepacific.com (Y.J.K.); hhkang@amorepacific.com (H.H.K.)

**Keywords:** antioxidant effect, *Camellia japonica*, reactive oxygen species

## Abstract

The aim of this study was to investigate the antioxidant properties of the ethanol extract of the flower of *Camellia japonica* (*Camellia* extract). *Camellia* extract exhibited 1,1-diphenyl-2-picrylhydrazyl radical and intracellular reactive oxygen species (ROS) scavenging activity in human HaCaT keratinocytes. In addition, *Camellia* extract scavenged superoxide anion generated by xanthine/xanthine oxidase and hydroxyl radical generated by the Fenton reaction (FeSO_4_ + H_2_O_2_) in a cell-free system, which was detected by electron spin resonance spectrometry. Furthermore, *Camellia* extract increased the protein expressions and activity of cellular antioxidant enzymes, such as superoxide dismutase, catalase and glutathione peroxidase. These results suggest that *Camellia* extract exhibits antioxidant properties by scavenging ROS and enhancing antioxidant enzymes. *Camellia* extract contained quercetin, quercetin-3-*O*-glucoside, quercitrin and kaempferol, which are antioxidant compounds.

## 1. Introduction

Oxidative damage initiated by reactive oxygen species (ROS) is a major contributor to the aging process [1]. Skin is a major candidate and target of oxidative stress, and during the skin aging process, ROS levels rise and antioxidant defenses decline [2]. Due to the high prevalence of potential biological targets for oxidative damage, skin is very susceptible to such reactions. For example, skin is rich in lipids, proteins, and DNA, all of which are extremely sensitive to the oxidation process [3]. The antioxidant system is an important defense mechanism against oxidative cell damage [4]. The antioxidant system is consisted of intrinsic enzymes, including superoxide dismutase (SOD), catalase (CAT), glutathione peroxidase (GPx), glutathione reductase, and extrinsic antioxidant nutrients, which serve to reduce free radicals to less toxic states [5]. Some studies suggest that age-related decrease in antioxidant enzyme activity is consistent with increased free radical damage that contributes to aging [6].

*Camellia japonica* is a broad-leaved evergreen woody species, widely distributed in Japan and Korea. The flowers and flower buds of *Camellia japonica* have been used for the treatment of bleeding and as an anti-inflammation in oriental traditional medicine [7]. Studies have been conducted on the constituents of *Camellia japonica*, including saponins in fruits [8] and seeds [9], flavonol glycosides in leaves [10], and triterpenes, flavonols and phenolic compounds in flowers [11,12]. However, the biological effects of *C. japonica* have been less frequently studied. In the present study, we focused on investigating the *in vitro* antioxidant effect of the ethanol extract from flower of *Camellia* extract in human keratinocytes.

## 2. Results

**Figure 1.**
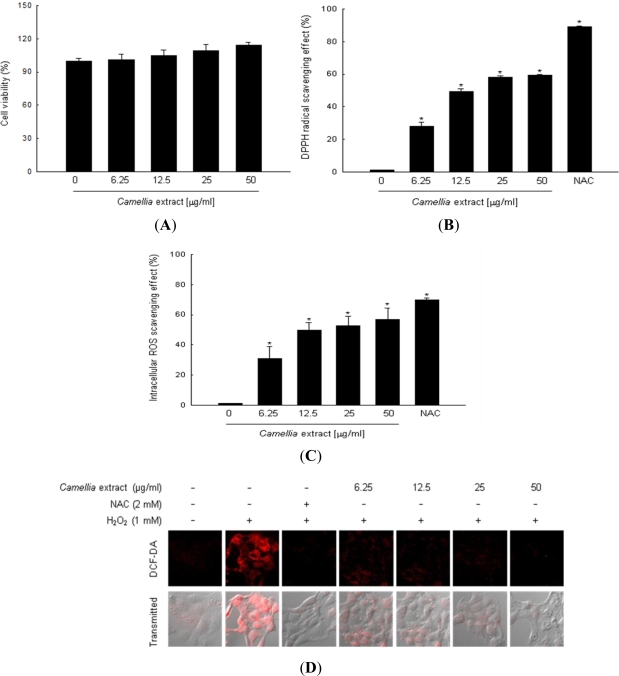
The effects of *Camellia* extract on cytotoxicity, scavenging DPPH radicals and intracellular ROS. **(A)** Cells were treated with various concentrations of *Camellia* extract. Cell viability was determined 24 h later by MTT assay. The data represent three experiments and are expressed as mean ± SE; **(B)** The amount of DPPH radical was determined spectrophotometrically at 520 nm; **(C)** Intracellular ROS generated was detected using a spectrofluorometer after DCF-DA staining; **(D)** Representative confocal images illustrate the increase of red fluorescence intensity of DCF produced by ROS in H_2_O_2_ treated cells compared to control. * Indicates significantly different from control (*p* < 0.05).

*Camellia* extract did not show the cytotoxicity to human HaCaT keratinocytes at 6.25 μg/mL, 12.5 μg/mL, 25 μg/mL, and 50 μg/mL (Figure 1A). *Camellia* extract scavenged DPPH radical, 28% at 6.25 μg/mL, 49% at 12.5 μg/mL, 58% at 25 μg/mL, and 60% at 50 μg/mL compared to 89% at 2 mM of NAC used as positive control (Figure 1B). Fluorescence spectrometric data revealed that intracellular ROS scavenging activity of *Camellia* extract was consistent with its DPPH radical scavenging activity, 31% at 6.25 μg/mL, 50% at 12.5 μg/mL, 53% at 25 μg/mL, and 57% at 50 μg/mL compared to 70% at 2 mM of NAC (Figure 1C). The fluorescence intensity of DCF-DA staining was also measured using confocal microscope. Analysis of confocal microscope showed that *Camellia* extract reduced the red fluorescence intensity upon H_2_O_2_ treatment, thus reflecting a reduction of ROS generation (Figure 1D).

**Figure 2.**
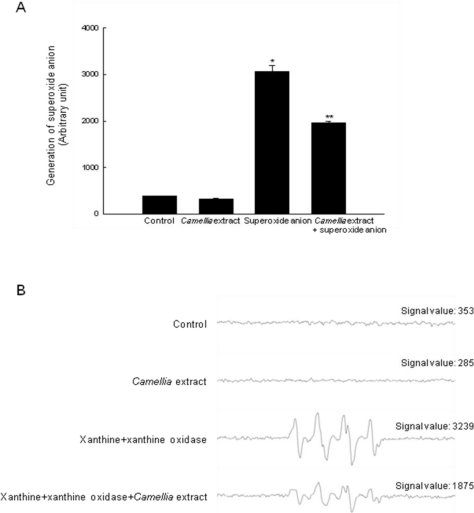
The scavenging effect of *Camellia* extract against superoxide anion. Superoxide anion generated by xanthine and xanthine oxidase reacted with DMPO, and the resultant DMPO/.OOH adducts were detected by ESR spectrometry. Results are shown in **(A)** histogram (mean ± SE) and **(B)** representative peak data. * Indicates significantly different from control (*p* < 0.05).

We chose 50 μg/mL as the optimal dose of *Camellia* extract for further investigations. Control or *Camellia* extract at 50 μg/mL showed no specific signal of superoxide anion, while in the xanthine/xanthine oxidase system, superoxide anion signal increased to 3239. *Camellia* extract treatment decreased superoxide anion signal in the xanthine/xanthine oxidase system to 1875 (Figure 2A and B).

**Figure 3.**
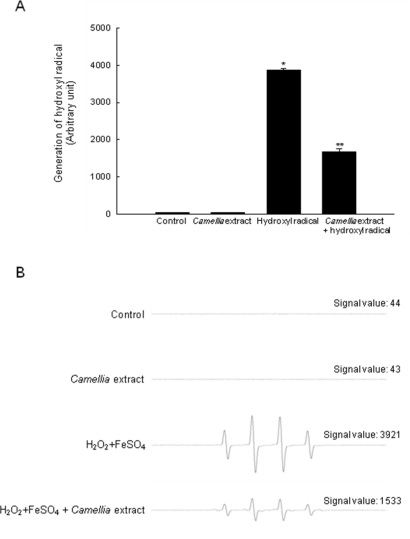
The scavenging effect of *Camellia* extract against hydroxyl radical. Hydroxyl radical generated by the Fenton reaction (H_2_O_2_+FeSO_4_) reacted with DMPO, and the resultant DMPO/.OH adducts were detected by ESR spectrometry. Results are expressed in **(A)** histogram (mean ± SE), and **(B)** representative peak data. * Indicates significantly different from control (*p* < 0.05).

Similarly, *Camellia* extract decreased generation of hydroxyl radical in the FeSO_4_ + H_2_O_2_ system from 3921 to 1533 (Figures 3A and B). Taken together, these results suggest that *Camellia* extract can directly scavenge ROS.

**Figure 4.**
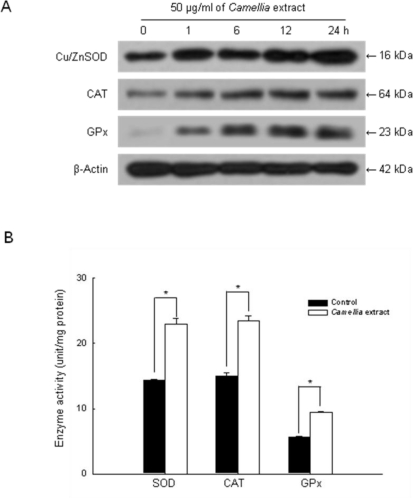
The effects of *Camellia* extract on protein expression and antioxidant enzyme activity. **(A)** SOD, **(B)** CAT and **(C)** GPx activity is expressed as unit per mg protein. * Indicates significantly different from control (*p* < 0.05).

In order to investigate whether the radical scavenging activity of *Camellia* extract was mediated by antioxidant enzyme activities, the protein expressions and activities of SOD, CAT and GPx in *Camellia* extract-treated cells were measured. As shown in Figure 4A, *Camellia* extract increased the protein expressions of these antioxidant enzymes in a time-dependent manner. At 24 h, the SOD activity with *Camellia* extract demonstrated 22.9 U/mg of protein, compared to 14.3 U/mg of protein in the control (Figure 4B, left). With respect to CAT, *Camellia* extract increased 23.4 U/mg of protein at 24 h, compared to 15.0 U/mg of protein in the control (Figure 4B, middle). Finally, GPx activity in *Camellia* extract-treated cells was also significantly increased to 9.4 U/mg of protein at 24 h, compared to 5.6 U/mg of protein in the control (Figure 4B, right). These results suggest that *Camellia* extract can increase the protein expressions and activities of antioxidant enzymes, such as SOD, CAT and GPx.

**Figure 5.**
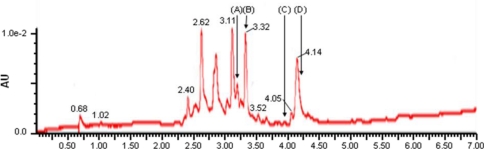
LC-MS/MS chromatogram of flavonoids from *Camellia* extract. Each peak indicates **(A)** quercetin-3-*O*-glucoside, **(B)** quercitrin, **(C)** quercetin, and **(D)** kaempferol.

And LC-MS/MS chromatogram of *Camellia* extract showed quercetin, quercetin-3-*O*-glucoside, quercitrin (a glycoside rhamnose of quercetin) and kaempferol, which are antioxidant compounds (Figure 5).

## 3. Discussion

The antioxidant properties of *Camellia* extract were evaluated in two categories: direct action on superoxide and hydroxyl radical scavenging in a cell-free system, and indirect action through induction of antioxidant enzymes. *Camellia* extract exerted direct scavenging activity on superoxide and hydroxyl radical as shown by ESR spectrometry. It was reported that the flower of *C. japonica* contained flavonols, such as quercetin, kaempferol, and sexangularetin, and phenolic compounds, such as *p*-hydroxybenzoic acid, protocatechuic acid and gallic acid [12]. The antioxidant compounds of *Camellia* extract in our study contained quercetin, quercetin-3-*O*-glucoside, quercitrin (a glycoside rhamnose of quercetin) and kaempferol. Quercetin exerts its antioxidant activity through scavenging ROS and preventing ROS formation with chelating transition metal ions, such as iron and copper [13,14]. Quercitrin has a free radical scavenging activity in a model of auto-oxidation of rat cerebral membranes [15]. Kaempferol has shown strong inhibitory/scavenging activity on ROS generation with numerous hydroxyl groups on their structures [16]. Moreover, it has been found to be a particularly potent blocker of extracellular ROS production, and to inhibit the ascorbate-dependent NADH oxidase and superoxide anion production activities of the neuronal plasma membrane redox chain [17]. Therefore, the ROS scavenging effect of *Camellia* extract demonstrated in this study might have been associated with flavonol or phenolic compounds.

Antioxidant enzymes, such as SOD, CAT, and GPx are the main antioxidant defense system. SOD plays an important role in scavenging superoxide anion which are formed during the early stages of oxidative stress, and in preventing aging [18]. SOD catalyzes the conversion of superoxide to hydrogen peroxide plus dioxygen. SOD can be classified into three groups, Cu/Zn SOD, Mn SOD, and Fe SOD, by the metals that they contain at their active sites. Cu/Zn SOD is usually found in the cytoplasm of eukaryotic cells and Mn SOD in mitochondria, whereas prokaryotic cells contain Fe SOD and Mn SOD [19]. Hydrogen peroxide is a harmful by-product of many normal metabolic processes. However, CAT and GPx are frequently used by cells to rapidly catalyze the decomposition of hydrogen peroxide [20]. CAT catalyzes the formation of water and oxygen from hydrogen peroxide and prevents oxidative damage [21]. GPx is a well-known selenoenzyme that functions as an antioxidant, and catalyzes the reduction of hydroperoxides, including hydrogen peroxides, by reduced glutathione and protect cells from oxidative damage [22].

**Figure 6.**
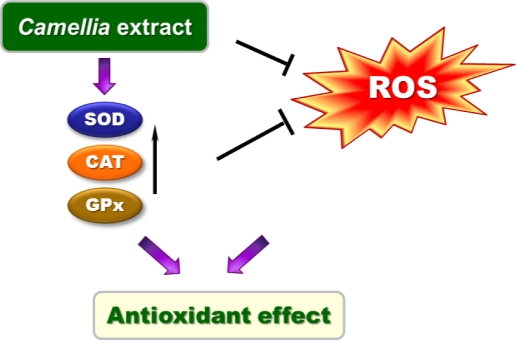
A proposed antioxidant action of *Camellia* extract, which explains scavenging of ROS and the enhancing effect of antioxidant enzymes.

Quercetin has been reported to reduce or prevent oxidative stresses induced by ultraviolet A through activation of antioxidant enzymes and induction of their expressions [23]. Also, quercetin has antioxidant and cytoprotective properties against renal ischemia-reperfusion injury by inducing SOD, CAT, GPx expression and activating their activities [24]. Kaempferol enhances the antioxidant properties by raised antioxidant proteins expression, such as metallothionein, CAT, and SOD [25]. Moreover, kaempferol inhibits H_2_O_2_-induced lipid peroxidation and enhances the activity of SOD and CAT [26,27]. Quercitrin enhances intracellular antioxidant defense against free radicals by increasing production of antioxidant enzymes [28]. *Camellia* extract increased the activity and protein levels of these enzymes, which may mediate its inhibition of ROS production. Thus, *Camellia* extract not only directly quenches ROS, but also indirectly induces antioxidant enzymes. In conclusion, *Camellia* extract exerts *in vitro* its antioxidant properties by scavenging ROS and enhancing antioxidant enzymes activities (Figure 6).

## 4. Experimental Section

### 4.1. Reagents

*Camellia* extract was obtained from the Amore Pacific Company (Gyeonggi-do, Korea). The UPLC-MS profile of *Camellia* extract was obtained from Jeju bio-industry development center of Jejutechnopark (Jeju, Korea). 1,1-Diphenyl-2-picrylhydrazyl (DPPH) radical, *N*-acetyl cysteine (NAC), 5,5-dimethyl-1-pyrroline-*N*-oxide (DMPO) and 2’,7’-dichlorodihydrofluorescein diacetate (DCF-DA) were purchased from the Sigma chemical company (St. Louis, MO, USA). Cu/Zn SOD and CAT antibodies were purchased from the Biodesign international company (Saco, Maine, USA). GPx antibody was purchased from Santa Cruz biotechnology (Delaware Avenue, CA, USA). All other chemicals and reagents used were of analytical grade.

### 4.2. Cell Culture

The HaCaT (human keratinocyte) cells were maintained at 37 °C in an incubator, at a humidified atmosphere of 5% CO_2_, and cultured in Dulbecco’s modified Eagle’s medium containing 10% heat-inactivated fetal calf serum, streptomycin (100 μg/mL) and penicillin (100 U/mL).

### 4.3. Cell Viability Assay

Cells were seeded in a 96 well plate at a concentration of 1 × 10^5^ cells/mL, and after plating for 16 h, *Camellia* extract at 6.25, 12.5, 25, and 50 μg/mL was added to the plate. After incubating for 24 h at 37 °C, fifty microliter of MTT stock solution (2 mg/mL) was added to each well of a total reaction volume of 200 μL. After incubating for 4 h, the plate was centrifuged at 800 × g for 5 min and the supernatants aspirated. The formazan crystals in each well were dissolved in 150 μL dimethylsulfoxide and the absorbance at 540 nm read on a scanning multi-well spectrophotometer.

### 4.4. DPPH Radical Scavenging Activity

*Camellia* extract at 6.25, 12.5, 25, and 50 μg/mL (10 μL) were added to a 1 × 10^−4^ M solution of DPPH (190 μL) in methanol. The resulting reaction mixture was shaken vigorously. After 3 h, the amount of remaining DPPH was determined at 520 nm using a spectrophotometer.

### 4.5. Intracellular ROS Scavenging Activity

The DCF-DA method was used to detect the intracellular ROS levels [29]. Cells were seeded 1.5 × 10^5^ cells/well and 16 h after plating, cells were treated with *Camellia* extract at 6.25, 12.5, 25, and 50 μg/mL (10 μL). After 30 min, 1 mM of H_2_O_2_ (10 μL) was added to the plate. Cells were incubated for an additional 30 min at 37 °C. After the addition of 25 μM of DCF-DA solution for 10 min, fluorescence of 2’,7’-dichlorofluorescein was detected using a PerkinElmer LS-5B spectrofluorometer. Image analysis for the generation of intracellular ROS was achieved by seeding cells on a cover-slip loaded six well plate at 2 × 10^5^ cells/well. Sixteen hours after plating, cells were treated with *Camellia* extract. One hour later, 1 mM of H_2_O_2_ was added to the plate. Twenty four hours later, 100 μM DCF-DA was added to each well and the cells were incubated for an additional 30 min at 37 °C. After washing with phosphate buffered saline (PBS), the stained cells were mounted onto a microscope slide in mounting medium (DAKO, Carpinteria, CA, USA). Images were collected using the laser scanning microscope 5 PASCAL program (Carl Zeiss, Jena, Germany) on a confocal microscope.

### 4.6. Detection of Superoxide Anion

Superoxide anion was generated by xanthine and xanthine oxidase, which were then quickly reacted with a nitrone spin trap, DMPO. The resultant DMPO/·OOH adducts were detected using an electron spin resonance (ESR) spectrometer. The ESR spectrum was recorded using JES-FA ESR spectrometer (JEOL, Tokyo, Japan), at 2.5 min after being mixed in a phosphate buffer solution (pH 7.4) with 20 μL of 3 M DMPO, 20 μL of 10 mM xanthine, 20 μL of 0.25 U xanthine oxidase, and 50 μg/mL of *Camellia* extract. The parameters of the ESR spectrometer were set at the following conditions: magnetic field of 336.5 mT, power of 5.00 mW, frequency of 9.4380 GHz, modulation amplitude of 0.2 mT, gain of 200, scan time of 0.5 min, scan width of 10 mT, time constant of 0.03 sec, and a temperature of 25 °C.

### 4.7. Detection of Hydroxyl Radical

Hydroxyl radicals were generated by the Fenton reaction (H_2_O_2_+FeSO_4_), which were then quickly reacted with DMPO. The resultant DMPO/^.^OH adducts were detected using an ESR spectrometer. The ESR spectrum was recorded using JES-FA ESR spectrometer at 2.5 min after being mixed in a phosphate buffer solution (pH 7.4) with 0.2 ml of 0.3 M DMPO, 0.2 mL of 10 mM FeSO_4_, 0.2 mL of 10 mM H_2_O_2_, and 50 μg/mL of *Camellia* extract. The parameters of the ESR spectrometer were set at the following conditions: magnetic field of 336.5 mT, power of 1.00 mW, frequency of 9.4380 GHz, modulation amplitude of 0.2 mT, gain of 200, scan time of 0.5 min, scan width of 10 mT, time constant of 0.03 sec, and a temperature of 25 °C [30].

### 4.8. Western Blot Analysis

Cells were lysed in 100 μL of a lysis buffer [120 mM NaCl, 40 mM Tris (pH 8), 0.1% NP 40]. Aliquots of the lysates (40 μg of protein) were boiled for 5 min and electrophoresed in 10% SDS-polyacrylamide gel. The blots in the gels were transferred onto nitrocellulose membranes (Bio-Rad, Hercules, CA, USA), and subsequently incubated with anti-primary antibodies. The membranes were further incubated with secondary anti-immunoglobulin-G-horseradish peroxidase conjugates (Pierce, Rockford, IL, USA), followed by exposure to X-ray film. The protein bands were detected using an enhanced chemiluminescence western blotting detection kit (Amersham, Little Chalfont, Buckinghamshire, UK).

### 4.9. Measurement of SOD Activity

Cells were seeded in a culture dish at 1 × 10^5^ cells/mL, and were treated with *Camellia* extract at 50 μg/mL. After 1 h, 150 μM of H_2_O_2_ was added to the plate, which was incubated for a further 24 h. The harvested cells were suspended in 10 mM phosphate buffer (pH 7.5) and then lysed on ice by sonicating twice for 15 sec. Triton X-100 (1%) was then added to the lysates and was incubated for 10 min on ice. The lysates were centrifuged at 5,000 × g for 10 min at 4 °C to remove the cellular debris. Fifty micrograms of protein was added to 500 mM phosphate buffer (pH 10.2) and 1 mM epinephrine. Epinephrine rapidly undergoes auto-oxidation at pH 10 to produce adrenochrome, a pink-colored product, which was assayed at 480 nm using an ultraviolet/visible spectrophotometer in kinetic mode. SOD inhibits auto-oxidation of epinephrine. The rate of inhibition was monitored at 480 nm and the amount of enzyme required to produce 50% inhibition was expressed as units/mg protein.

### 4.10. Measurement of CAT Activity

Fifty milligrams of protein was added to 50 mM phosphate buffer (pH 7) containing 100 mM H_2_O_2_. The reaction mixture was incubated for 2 min at 37 °C and the absorbance was monitored at 240 nm for 5 min. The change in absorbance with time was proportional to the breakdown of H_2_O_2_. The amount of enzyme required to breakdown 1 mM of H_2_O_2_ was expressed as units/mg protein.

### 4.11. Measurement of GPx Activity

Fifty milligrams of protein was added to 25 mM of phosphate buffer (pH 7.5) containing 1 mM EDTA, 1 mM NaN_3_, 1 mM glutathione, 0.25 units of glutathione reductase and 0.1 mM NADPH. After incubation for 10 min at 37 °C, H_2_O_2_ was added to the reaction mixture at a final concentration of 1 mM. Absorbance was monitored at 340 nm for 5 min. GPx activity was measured as the rate of NADPH oxidation by change in absorbance at 340 nm. The amount of enzyme required to oxidize 1 mM NADPH was expressed as units/mg protein.

### 4.12. Statistical Analysis

All the measurements were made in triplicate and all values are represented as means ± standard error (SE). The results were subjected to an analysis of the variance (ANOVA) using the Tukey test to analyze the difference. The p < 0.05 was considered significant.
